# Error-Related Neural Responses Recorded by Electroencephalography During Post-stroke Rehabilitation Movements

**DOI:** 10.3389/fnbot.2019.00107

**Published:** 2019-12-20

**Authors:** Akshay Kumar, Qiang Fang, Jianming Fu, Elena Pirogova, Xudong Gu

**Affiliations:** ^1^College of Engineering, Shantou University, Shantou, China; ^2^School of Engineering, RMIT University, Melbourne, VIC, Australia; ^3^2nd Hospital of Jiaxing, Jiaxing, China

**Keywords:** assist-as-needed, brain–computer interface (BCI), electroencephalography (EEG), error-related potential (ErrP), stroke rehabilitation

## Abstract

Error-related potential (ErrP) based assist-as-needed robot-therapy can be an effective rehabilitation method. To date, several studies have shown the presence of ErrP under various task situations. However, in the context of assist-as-needed methods, the existence of ErrP is unexplored. Therefore, the principal objective of this study is to determine if an ErrP can be evoked when a subject is unable to complete a physical exercise in a given time. Fifteen stroke patients participated in an experiment that involved performing a physical rehabilitation exercise. Results showed that the electroencephalographic (EEG) response of the trials, where patients failed to complete the exercise, against the trials, where patients successfully completed the exercise, significantly differ from each other, and the resulting difference of event-related potentials resembles the previously reported ErrP signals as well as has some unique features. Along with the highly statistically significant difference, the trials differ in time-frequency patterns and scalp distribution maps. In summary, the results of the study provide a novel basis for the detection of the failure against the success events while executing rehabilitation exercises that can be used to improve the state-of-the-art robot-assisted rehabilitation methods.

## Introduction

Stroke is the second leading cause of death and the third leading cause of disability in the world (Johnson et al., 2016). Depending on the extent and the location of damage due to stroke, stroke survivors suffer from a various degree of body functionality impairment (Kalaria et al., 2016). The most notable impairments include cognitive impairment, dementia, and limb movement impairment (Kalaria et al., 2016). Nevertheless, recovery from the disability due to stroke is possible through neuroplasticity (Basteris et al., 2014). Neuroplasticity is brain’s natural process aimed to reorganize itself by forming new neural connections, especially in response to learning or experiences that result in partial recovery from the disability. Post-stroke rehabilitation accelerates this process of neurological changes and ultimately helps in attaining a higher rate of recovery (Liu et al., 2017).

Motor function impairment affects the patient’s activities of daily living (ADLs) (Basteris et al., 2014). Therefore, recovery from motor disabilities is one of the primary objectives of the post-stroke rehabilitation program. Historically, in post-stroke rehabilitation, the patients are asked to perform certain physical exercises using their affected limb (Liu et al., 2017). Previous studies have described the role of intensive and repetitive rehabilitation exercises in promoting the rate of recovery from motor disabilities (Grosmaire and Duret, 2017; Liu et al., 2017; Tacchino et al., 2017). In addition, several studies have highlighted the significance of the active participation of the patient in performing rehabilitation exercises to promote motor recovery (Grosmaire and Duret, 2017; Tacchino et al., 2017). However, the key issue is that stroke patients cannot perform the rehabilitation exercises repetitively and actively due to their motor impairment (Basteris et al., 2014; Yue et al., 2017).

Assist-as-needed (AAN) robot therapy-based rehabilitation program helps in eliminating the issues mentioned above and allows the patients to perform exercises repetitively and actively (Basteris et al., 2014; Grosmaire and Duret, 2017). In AAN based robot therapies, assistance is provided to the patient in performing the rehabilitation exercise when he/she is unable to perform it on his/her own and *vice versa*. A few well-known EEG based BCI approaches, including movement-related cortical potentials (MRCP) (Liu et al., 2017), event-related synchronization/desynchronization (ERS/ERD) (Liu et al., 2017) and surface-electromyographic (sEMG) signals (Basteris et al., 2014), are most popular in developing assistive exoskeletons that assist stroke survivors in performing physical exercises. Error-related potential (ErrP) is another event-related potential (ERP) signal which is gaining popularity recently in BCI research due to its inherent intrinsic human feedback mechanism (Chavarriaga et al., 2014). It is an ERP that is elicited when a human perceives an error (Chavarriaga et al., 2014).

The existing body of research on ErrP suggests that ErrP signal is elicited under certain task situations. Response ErrP occurs when the subject is asked to respond as quickly as possible (e.g., choice reaction task) (Olvet and Hajcak, 2009). Feedback ErrP occurs when the subject realizes an error upon given feedback of the task (Lopez-Larraz et al., 2010). Interaction ErrP occurs when the subject is interacting with a machine, and the machine misinterpreted an instruction given (Ferrez and Del R. Millán, 2008). Observation ErrP occurs when the subject recognizes an error made by a machine or external system (Roset et al., 2014). Recently, three new types of errors, namely target, outcome, and execution ErrPs have been reported (Milekovic et al., 2013). To date, a number of studies have investigated the ErrP signal and its applicability in the EEG-based BCIs (Olvet and Hajcak, 2008; Chavarriaga et al., 2014; Weinberg et al., 2015; Kim et al., 2017). In Omedes et al. (2018), authors developed a 3D virtual interface that simulated participants’ hand to reach and grasp different virtual objects and found that an ErrP evokes in erroneous commands. In Kim et al. (2017), ErrP was employed to detect if a robot has made an error in recognizing the gesture made by the participant. In Yazmir and Reiner (2017), authors developed a virtual tennis game in which a difference in EEG response was observed when the participant successfully hit to that of miss the target.

Although significant research has been carried out on ErrP, there is no single study which discusses the feasibility of ErrP in implementing assist-as-needed robot therapy approach. With the aim of filling the gap mentioned above in BCI based methods, in this study we investigated if an ErrP signal is evoked when a participant is unable to perform a physical exercise. Fifteen stroke patients participated in this EEG experiment in which they performed a standard rehabilitation exercise. We hypothesized that a difference similar to error-related potentials will exist when the stroke patients are unable to perform a rehabilitation exercise (failure trial) to that of when they complete the same rehabilitation exercise (success trial) in a given time. Results of this study will provide a novel basis for the BCI based methods to be implement in the AAN based robot therapy, hence forming a foundation of a new type of task situation in which the ErrP brain signal can be elicited. This will assist in differentiating a failure trial against a success trial in performing a rehabilitation exercise. The developed ErrP based brain-in-the-loop approach is expected to enhance the efficiency of robot-based stroke rehabilitation programs.

## Materials and Methods

### Participants

Fifteen stroke patients (five females, mean age: 57.5 ± 11.3 years) participated in the experiment (see Table 1). The experiment was conducted in collaboration with the 2nd Hospital of Jiaxing, China. All participants voluntarily took part in the experiment and provided their written informed consent before commencement of experiments. The stroke patients’ affected limb’s impairment level was assessed using Brunnstrom stages of recovery by an expert panel from the 2nd Hospital of Jiaxing, China prior to experimentation (see Table 1). The study was approved by the Ethics Committee of the 2nd Hospital of Jiaxing, and the experiments were conducted in accordance with the declaration of Helsinki. The data of Patient 1 and 9 had to be excluded from the analysis because patient 1 aborted the experiment at the start and patient 9 was not performing the experiment as per the instructions given.

**TABLE 1:** Stroke patients’ information.

	**Range**	**Diagnosis**	**Affected**	**Days since**	**Brunnstrom**
**Patient**	**of age**		**side**	**stroke**	**stage**
1	50–55	Hemorrhage	Left	113	Stage II
2	75–80	Hemorrhage	Left	62	Stage IV
3	65–70	Infarction	Right	82	Stage II
4	55–60	Infarction	Right	11	Stage II
5	60–65	Infarction	Right	11	Stage II
6	30–35	Hemorrhage	Left	40	Stage I
7	45–50	Infarction	Right	31	Stage II
8	50–55	Hemorrhage	Right	41	Stage IV
9	60–65	Infarction	Left	53	Stage II
10	45–50	Infarction	Right	31	Stage IV
11	50–55	Infarction	Left	44	Stage III
12	70–75	Infarction	Left	48	Stage I
13	60–65	Hemorrhage	Right	18	Stage II
14	55–60	Infarction	Right	55	Stage II
15	55–60	Infarction	Right	43	Stage IV

*The data of patient 1 and 9 had to be excluded from the analysis because patient 1 aborted the experiment at the start and patient 9 was not performing the experiment as per the instructions given.*

### Task Description

Participants sat on a comfortable chair facing the LCD monitor (resolution of 1280 × 720 and refresh rate of 100 Hz) that delivered task instructions to the participants.

**FIGURE 1:**
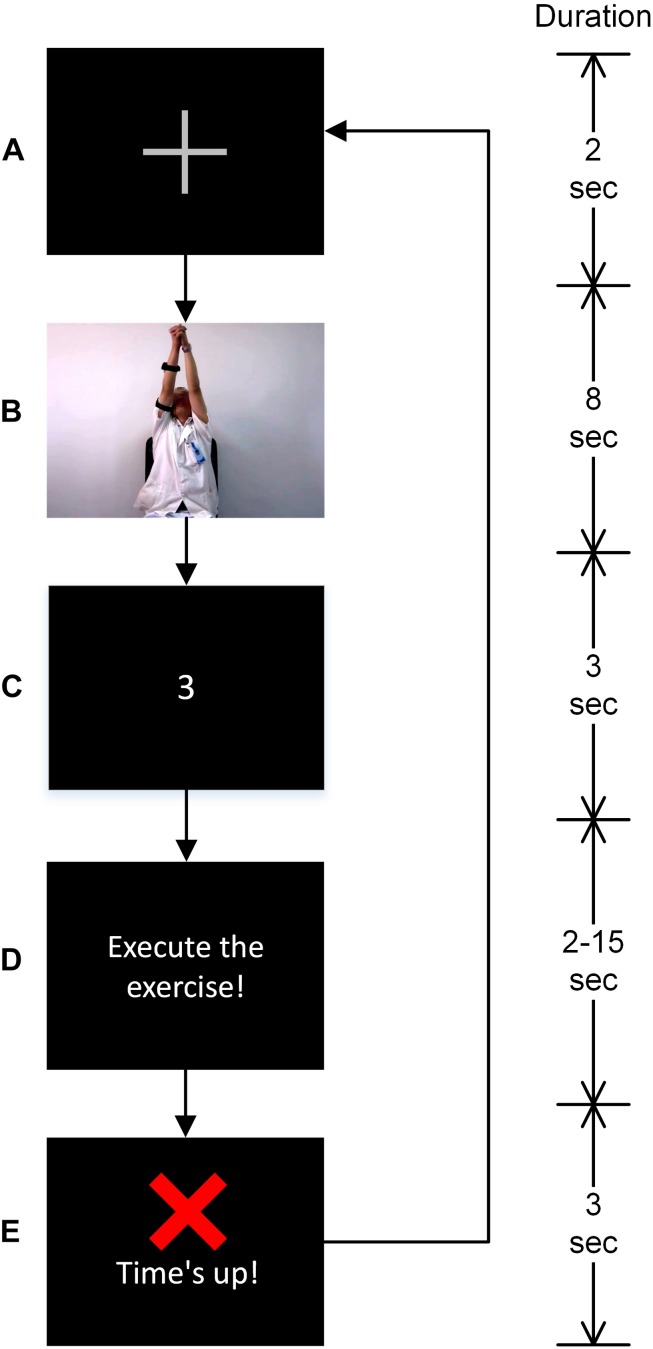
Description of the task’s trial and visual stimuli. Instructions were delivered in the following order. **(A)** A fixation cross marking the start of the trial, **(B)** exercise video, **(C)** a 3-2-1 timer, **(D)** image depicting instruction to start performing the exercise. The time given to perform the exercise varied between 2 and 15 s in a pseudo-random fashion and it was based on a preliminary investigation. **(E)** Participants were asked to complete the exercise before this screen appeared.

The experiment required participants to perform a standard Bobath’s rehabilitation exercise: shoulder flexion-extension while adjoining both hands. Already recorded video of a therapist performing the exercise was used to illustrate the know-how of the exercise to the participants. The video was collected from the 2nd Hospital of Jiaxing, China. Each participant was asked to participate in two sessions of the experiment including 24 trials each; however, patient 2, 3, 7, and 14 participated only in one session due to muscle fatigue. All participants’ data satisfied the minimum number of trials required to conduct an ErrP based ERP study, i.e., at least six trials of each class, as outlined in the previous studies (Olvet and Hajcak, 2009; Meyer et al., 2013). A fixation cross, as shown in Figure 1A appeared at the start of a trial. Followed by the fixation mark, the exercise video was played (see Figure 1B). Participants were asked to observe the exercise being described in the video. Afterward, a 3-2-1 timer (see Figure 1C) starts and the participants were asked to perform the exercise (accurately as depicted in the video) once the timer finishes and Figure 1D appears. Participants were given 2–15 s to complete the exercise. Roughly, in 35% of the trials, 2–4 s, in 10% of the trials 9–10 s and in the rest of the trials 15 s were given to complete the exercise in a pseudo-random fashion. Participants were asked to complete the exercise before the ‘Time’s up!’ screen (see Figure 1E) has appeared. Additionally, a beep sound was played on the onset of ‘Time’s up!’ screen for about 30 ms to inform the participants that the time has expired. The accuracy was preferred over the speed as the ErrP amplitudes are notably larger when the accuracy is emphasized over the speed (Gehring et al., 1993).

The time durations given to complete the exercise were based on a preliminary experiment such that the patients will not be able to complete the exercise in the short 2–4 s trials (named failure trials onwards) and will be able to successfully complete the exercise in the long 15 s trials (named success trials onwards). A complete exercise was marked in a trial when the participant started performing the exercise on the onset of the screen as in Figure 1D and execute the exercise depicted in the exercise video, and his/her arms come to rest before the onset of the screen as in Figure 1E. The medium 9–10 s trials were used to add additional uncertainty so that participants cannot anticipate trial durations. The medium 9–10 s trials were not included in the data analysis. Participants were asked to take rest for about 20 min in between two sessions to avoid muscle fatigue, and thus, the participants could keep full concentration in the experiment.

Experiment procedure was explained to the participants, and sufficient time was given for practice before the start of the experiment. The visual stimuli of the experiment were designed using Presentation software (version 20.2, Neurobehavioral Systems, Inc., Berkeley, CA, United States).

### Data Acquisition

Fifty-eight channels of monopolar EEG were recorded using an active EEG electrode system and g. HIamp amplifier (g. tec GmbH, Austria) as per the international 10–20 system. Electro-oculogram (EOG) signals were recorded from the electrodes placed above and below the left eye (FP1, VEOG) and on the outer canthi of both eyes (F9, F10). The EOG data were recorded to remove the eye-blinks artifacts from the EEG signals. Channels were referenced to the right earlobe and grounded to AFz location. Bipolar sEMG signals were recorded from the deltoid posterior muscle. All electrodes impedance was kept below 10 kΩ. All three physiological signals were sampled at 2400 Hz with a 50 Hz notch filter. EEG and EOG data were additionally filtered with 0.01–1000 Hz band-pass filter, and EMG data were additionally filtered with a 10–500 Hz band-pass filter to remove the slow drift and high-frequency noises in the signal (Farquhar and Hill, 2013).

### Data Analysis

To prepare the raw data for further analysis, all the recorded physiological signals were preprocessed offline. Data preprocessing was performed in MATLAB and EEGLAB toolbox (Delorme and Makeig, 2004). The preprocessing steps are as follows:

(1) High-pass filtered the data to remove low-frequency drifts using Windowed sinc finite impulse response filter (zero phase shift), 0.1 Hz cut-off frequency, as per the parameters recommended in Widmann et al. (2015).
(2) Low pass filtered the data using Windowed sinc finite impulse response filter (zero phase shift), 128 Hz cut-off frequency, as per the parameters recommended in Widmann et al. (2015).
(3) Data were down-sampled to 512 Hz.
(4) Artifactual channels were removed with manual inspection of the data.
(5) Artifact subspace reconstruction (ASR) (Mullen et al., 2013) method was used to remove transients, stereotypical and non-stereotypical high amplitude artifacts from the continuous EEG data with parameters [standard deviation (SD) 20, sliding window length 500 ms and correlation coefficient 0.8] as suggested in Chang et al. (2018).
(6) Independent component analysis was used to remove any artifactual component left in the data (Delorme and Makeig, 2004). The components that comprised of ocular, cardiac and muscular artifacts were removed. A hybrid approach constituting ICLabel (EEGLAB) and visual inspection was used to filter out the artifactual components.
(7) Data were segmented into epochs ranging from −200 to 800 ms relative to the onset of Figure 1E. Further analysis was performed on the extracted epochs.
(8) Data epochs with maximum signal amplitude exceeding ± 75 μV and improbability exceeding 6 SD for single channels and 2 SD for all channels were flagged as outliers and were removed; about 5% of the trials were removed (Sörnmo and Laguna, 2005; Delorme et al., 2007).

Teager Kaiser energy operator (TKEO) method is a powerful method to detect the onset and offset of the muscle activity from the sEMG signal (Li et al., 2007). The discrete TKEO operator ψ is defined as:

$$\Psi \,\left[x\,\left(n\right)\right]=x^{2}\,\left(n\right)-x\,\left(n+1\right)\,x\,\left(n-1\right)$$

where *x* is the sEMG value and *n* is the sample number (Li et al., 2007).

Muscle activity onset was considered when the TKEO operator ψ value exceeds a fixed threshold α for more than 25 consecutive samples, and similarly muscle activity offset was considered when the ψ value remains below the α threshold for more than 25 consecutive samples after the onset of the muscle activity (Solnik et al., 2008). The threshold α is defined as:

$$\alpha =\mu_{0}+j\,\delta_{0}$$

where μ_0_ and δ_0_ are mean and SD of the background sEMG noise in the TKEO domain, whereas *j* is a scale factor for δ_0_. Value of *j* was chosen to be 7 based on the recommendation in Li et al. (2007). In a trial, if the participant started performing the exercise before Figure 1D screen appeared or in other words if the onset of the muscle activity was before the Figure 1D screen appeared, it was removed. The trials in which patients failed to observe the onset of Figure 1D screen and consequently delayed the execution of exercise were removed as well. Furthermore, the authenticity of failure and success trials were confirmed with the offset of the muscle activity, i.e., in failure trials, muscle activity offset should occur after the onset of the ‘Time’s up!’ screen and in success trials it should occur before the onset of the ‘Time’s up!’ screen. All the trials that had ambiguity in being a success or failure trials and the trials in which the participant did not perform the exercise at all were removed. Overall less than 20% of the trials were rejected. The artifacts free unambiguous success and failure trials were considered for further analysis.

Individual failure and success related ERP waveforms were calculated by averaging over the success and failure epochs of the same individual. Grand-average success and failure ERP waveforms were calculated by averaging over individual success and failure ERPs. Difference of individual as well as grand-average failure and success ERP waveforms were calculated further and were smoothed out with 25 Hz low-pass FIR filter for illustration purpose and statistical analysis. Topographical scalp maps provide reference-bias-free multi-channel information of the EEG signals (Murray et al., 2008). Therefore, scalp maps were also calculated using the grand-average success and failure EEG signals on the most prominent peaks.

In order to gauge induced and evoked power in the ERP signal, time-frequency analysis was performed (Dickter and Kieffaber, 2014). Evoked power is phase-locked to the stimulus presented; however, induced power (induced oscillatory activities) is not phase-locked and hence get canceled out in averaging (Dickter and Kieffaber, 2014). Nevertheless, both evoked and induced power are time-locked to the stimulus event (Dickter and Kieffaber, 2014). Event-related spectral perturbations (ERSPs) represents amplitude dynamics in a broad range of frequencies as a function of time furnishing information of both evoked and induced power unlike the grand-average ERPs that only provide information of the evoked power (Dickter and Kieffaber, 2014). On the other hand, Inter-trial coherence (ITC) computes phase synchronization across trials at each time point in a broad range of frequencies (Dickter and Kieffaber, 2014). ITC ranges between zero and one where zero suggests random phases and one suggests perfectly coherent phases across trials (Dickter and Kieffaber, 2014). Thus, localized changes in ERSP with a high value of ITC at that particular time and frequency represents evoked power whereas localized ERSP changes with low values of ITC represents induced power (Dickter and Kieffaber, 2014). Jointly with the ERSPs and the ITC, the power of the evoked response, as well as the induced oscillatory activities, can be gauged (Dickter and Kieffaber, 2014).

In result, time-frequency analysis was performed in EEGLAB to calculate the ERSPs and ITC with parameters as recommended in Delorme and Makeig (2004) and Dickter and Kieffaber (2014). ERSPs and ITC were calculated on full epoch length with a resolution of 4 ms and frequency resolution of 1 Hz approximately. Fast Fourier transform (FFT) with Hanning tapering was used for decomposition. The ERSP spectrogram post-0 ms was divided by the spectrogram before 0 ms for normalization. It is to be noted that the onset of Figure 1E screen is taken as 0 ms reference throughout the analysis. The significance of ERSPs deviation from the baseline was assessed using the bootstrap method (α = 0.05).

Statistical analysis was conducted using IBM SPSS Statistics 25 to estimate the statistical significance of the results. Statistical significance of the difference of failure and success ERP peaks’ amplitude (named difference ERP onwards) for each of the 13 participants were tested against zero with one-sample *t*-test (two-tailed). These individual difference ERP amplitudes were computed by averaging each patients’ failure and success trials separately and, then, extracting the amplitudes of the most prominent peaks and then taking a difference of that. The *t*-test null-hypothesis was that there is zero mean amplitude difference in the ERPs of success and failure trials, whereas the alternative hypothesis was non-zero mean amplitude difference. Previous studies have suggested that the ErrP signals have the highest activity around Cz and FCz electrodes (Chavarriaga and Millán, 2010; Spüler and Niethammer, 2015; Yazmir and Reiner, 2017). Therefore, the analysis was carried out on the most prominent peaks in the difference ERP at the Cz electrode location.

Shapiro–Wilk test (α = 0.05) was used to confirm the normal distribution of the mean samples before the statistical analysis (Shapiro and Wilk, 1965). The null-hypothesis of Shapiro–Wilk assumes a normal-distribution while alternative hypothesis denies that. Skewness and Kurtosis z-value were used as an additional measure to confirm the normality of the mean data samples (Cramer, 1998).

## Results

Strong evidence of differences in the EEG response of the success and the failure trials has been found. The obtained ErrP has some unique features pertaining to the novel task situation besides some features similar to the previously reported ErrP signal.

**FIGURE 2:**
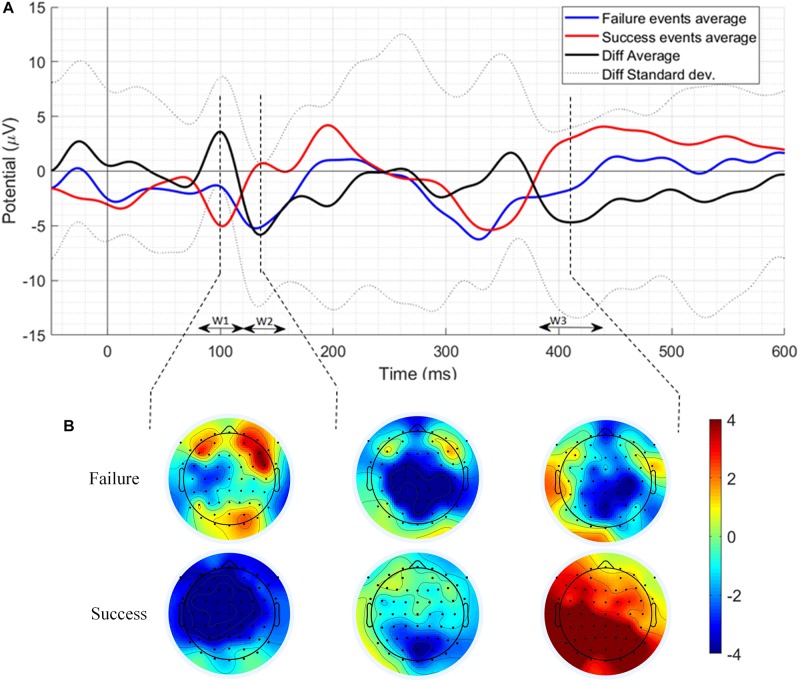
**(A)** The grand average ERPs of the failure events, success events, difference ERP along with its standard deviation at Cz electrode location. W1, W2, and W3 represent three times windows selected for analysis based on the most prominent peaks. **(B)** Scalp distribution maps of the most prominent peaks.

The result of the grand average ERPs of the failure and success trials and their difference at Cz location has been shown in Figure 2A. Three-time windows (W1: 80–120 ms, W2: 120–160 ms, and W3: 380–440 ms) were chosen for analysis based on the most prominent peaks. Failure events ERP has a positive component with a peak at 98 ms and an amplitude of −1.139 μV and also a negative component with a peak at 131 ms and an amplitude of −5.236 μV. In addition, a negative component related to failure events has been observed with a peak at 414 ms and an amplitude of −1.528 μV. Similarly, success events ERP has a negative component with a peak at 102 ms with an amplitude of −5.043 μV and also a positive component with a peak at 137 ms with an amplitude of 0.714 μV. Likewise, a positive component related to success events has been observed with a peak at 400 ms and an amplitude of 2.55 μV. Figure 2A also presents the results of the difference of ERPs of failure and success events (difference ERP) in the three time-windows along with its standard deviation. As can be seen in Figure 2A, in the difference ERP, a positive peak appears at 100 ms with an amplitude of 3.611 μV pursued by a negative peak at 135 ms with an amplitude of −5.827 μV followed by a prominent negative peak at 410 ms with an amplitude of −4.687 μV. The scalp maps of the grand-average success and failure ERPs have been showing a centro-parietal scalp distribution mainly as can be seen in Figure 2B.

**FIGURE 3:**
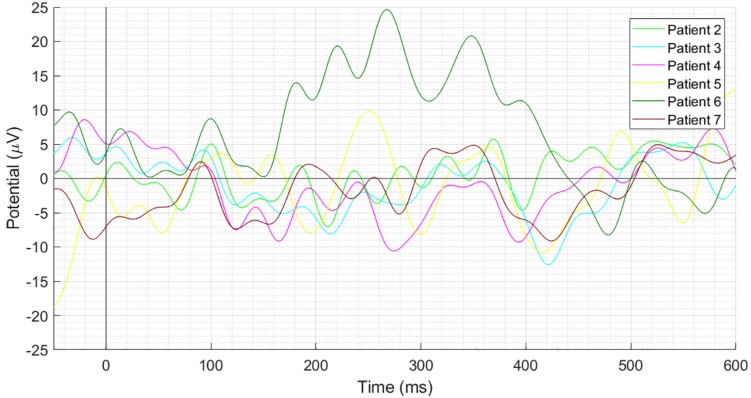
Individual difference ERPs at the Cz electrode for six patients. Results of only first six patients have been shown to prevent over-burdening the figure, while we got similar outcomes for each of the 13 patients, which can also be deduced from the SD shape exhibited in Figure 2A.

Figure 3 depicts individual waveforms of difference ERP. What stands out in Figure 3 is the repeating pattern of the individual difference ERPs. Results of only first six patients have been shown to prevent over-burdening the figure, while we got similar outcomes for each of the 13 patients, which can also be deduced from the SD shape exhibited in Figure 2A. Individual waveforms were based on all available epochs for each patient.

**FIGURE 4:**
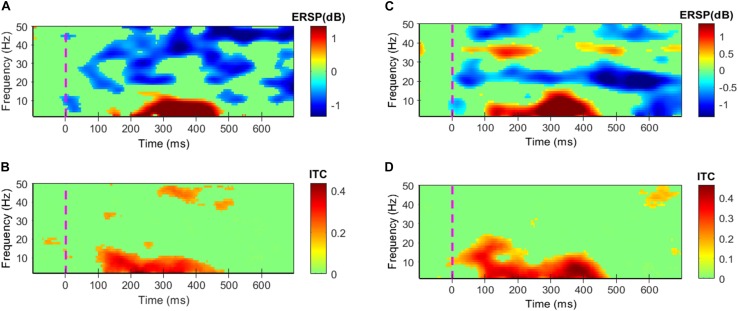
Grand average ERPs’ Event-related spectral perturbations (ERSPs) and inter-trial coherence (ITC). All colors except green shows statistically significant values at α = 0.05. **(A,B)** Depict ERSPs and ITC patterns for the failure events, respectively. **(C,D)** Depict ERSPs and ITC patterns for the success events respectively.

Event-related spectral perturbations and ITC of success and failure events have been depicted in Figure 4. The results showed in all four Figures 4A–D are statistically significant (α = 0.05); the insignificant areas are shown in the green color. Failure events associated evoked power is significantly high in the time-period 96–470 ms and 3–12 Hz, and ITC reaches up to 0.44 in this period as shown in Figure 4B. Induced power is significantly high in the time period 150–700 ms and 42–50 Hz, 640–700 ms and 11–24 Hz, 15–34 Hz at various times as shown in Figure 4A. Success events associated evoked power is significantly high in the time-period 100–460 ms and 3–10 Hz, 20–170 ms and 10–21 Hz and ITC reaches up to 0.46 in this period as shown in Figure 4D. Induced power is significantly high in the time period 550–700 ms and 3–22 Hz, 30–650 ms and 18–30 Hz, 34–38 Hz and 42–50 Hz at various times as shown in Figure 4C.

Shapiro–Wilk test results show that the assumption of normal distribution was not rejected for difference ERPs in all three-time windows at α = 0.05 (see Table 2). In addition, skewness and kurtosis z-value was within ± 1.96 for the three-time windows’ difference ERP peaks’ amplitudes that further strengthen the assumption of normal distribution.

**TABLE 2:** Shapiro–Wilk test *p-*value, mean, standard deviation, *t*-test statistics (two-tailed) result of the difference in difference ERP’s peak amplitudes against zero in the three time-windows.

	**Shapiro–Wilk**		***t*-Test statistics**
**Windows**	** test *p*-value**	**Mean (M) ± SD**	** (against zero)**
Window 1	0.073	4.509 ± 3.55	*t*(12) = 4.580, *p* = ≤ 0.001
Window 2	0.623	−6.556 ± 4.706	*t*(12) = −5.023, *p* = ≤0.001
Window 3	0.319	−6.406 ± 7.242	*t*(12) = −3.190, *p* = 0.008

One-sample *t*-test (two-tailed) was applied to examine if the amplitude of the difference ERP peak in the time window W1, W2, and W3 (see Figure 2A) significantly different against zero or not. The results of the statistical analysis are shown in Table 2. In window W1, the difference ERP’s positive peak amplitude is significantly higher than zero with *M* = 4.509, *SD* = 3.55, *t*(12) = 4.580, *p* = ≤ 0.001. In window W2, the difference ERP’s negative peak amplitude is significantly lower than zero with *M* = −6.556, *SD* = 4.706, *t*(12) = − 5.023, *p* = ≤0.001. In window W3, the difference ERP’s negative peak amplitude again significantly lower than zero with *M* = −6.406, *SD* = 7.242, *t*(12) = −3.190, *p* = 0.008.

**FIGURE 5:**
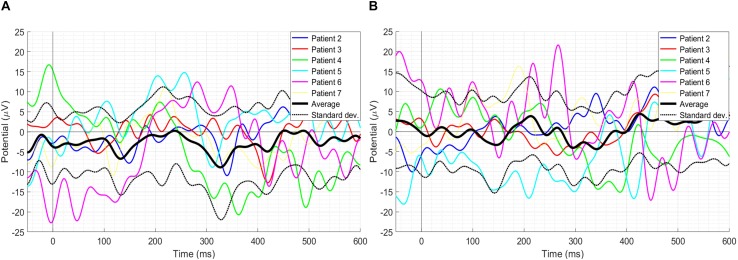
**(A)** The grand-average ERPs with its SD as well as individual ERPs of the 2-s failure trials. **(B)** The grand-average ERPs with its SD as well as individual ERPs of the 4-s failure trials. Results of only the first six patients have been shown to prevent over-burdening the figure, while we got similar outcomes for each of the 13 patients.

The literature on ErrP has highlighted several factors that affect its amplitude, such as speed and accuracy of task execution (Gehring et al., 1993, 2018). Thus, a step further, it has been investigated if the amount of exercise executed can affect the amplitude of failure events peaks in the three-time windows. It is understood that the failure trials in which 4 s were given to complete the exercise (named 4-s failure trials onwards), the amount of exercise executed was higher before the onset of ‘Time’s up!’ screen as there was more time in comparison to the failure trials in which 2 s were given to complete the exercise (named 2-s failure trials onwards). Therefore, the 2-s failure trials and the 4-s failure trials were separated and compared. The grand-average ERPs with its SD as well as the individual ERPs of 2- and 4-s failure trials at the Cz electrode location have been shown in Figures 5A,B respectively. Results of only first six patients have been shown to prevent over-burdening the figure, while we got similar outcomes for each of the 13 patients. Paired-samples *t*-test has been performed to evaluate the significance of the difference in the amplitudes of 2- and 4-s trials in the three-time windows. Shapiro–Wilk test (α = 0.05) was used to confirm the normal distribution of the mean samples before the statistical analysis, and the results have been shown in Table 3. Also, skewness and kurtosis z-value of the mean samples was within ± 1.96 for the three-time windows except for the 4-s failure trials skewness z-value in the window W2 which was −2.028. However, paired-samples *t*-test was used in this case as well to check any significant difference. The results of the statistical analysis are shown in Table 3. In window W1, the 2-s trials M and SD are −0.502 and 6.980 respectively and 4-s trials M and SD are 2.166 and 7.280 respectively; however, the difference is statistically insignificant with *t*(12) = −0.832, *p* = 0.422. In window W2, the 2-s trials M and SD are −8.366 and 7.258 respectively and 4-s trials M and SD are −3.679 and 10.801 respectively; nevertheless, the difference is statistically insignificant with *t*(12) = −1.161, *p* = 0.268. In window W3, the 2-s trials M and SD are −6.020 and 8.020 respectively and 4-s trials M and SD are −2.887 and 7.348 respectively; however, again the difference is statistically insignificant with *t*(12) = − 0.970, *p* = 0.351.

**TABLE 3:** Shapiro–Wilk test *p*-value, mean, standard deviation, paired samples *t*-test statistics (two-tailed) results of the difference in 2-s failure events’ peak amplitudes and 4-s failure events’ peak amplitudes in the three time-windows.

Windows	Type of failure event	Shapiro-Wilk test *p*-value	Mean ± SD	*t*-Test statistics (inter-)
Window 1	2-s trials	0.460	-0.502 ± 6.980	*t*(12) = -0.832, *p* = 0.422
	4-s trials	0.358	2.166 ± 7.280	
Window 2	2-s trials	0.935	-8.366 ± 7.258	*t*(12) = -1.161, *p* = 0.268
	4-s trials	0.113	-3.679 ± 10.801	
Window 3	2-s trials	0.281	-6.020 ± 8.020	*t*(12) = -0.970, *p* = 0.351
	4-s trials	0.076	-2.887 ± 7.348	

**FIGURE 6:**
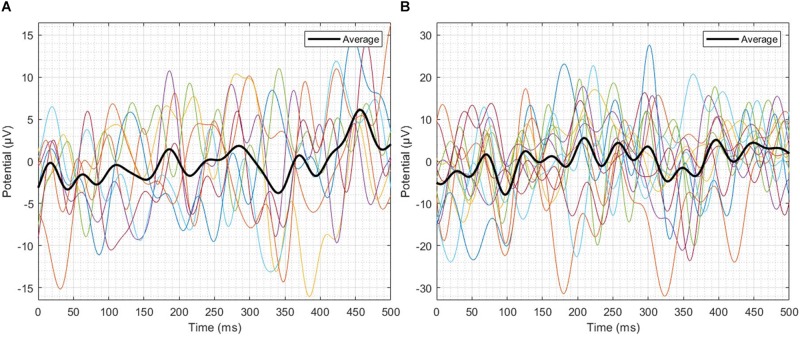
Single-trial EEG responses of patient 2. **(A)** Failure trials, **(B)** Success trials. In the three-time windows W1, W2 and W3, the average ITC of the failure trials has been observed to be 0.3486, and for the success trials, it has been observed to be 0.4187.

Single-trial EEG responses of patient 2 have been shown in Figure 6. To assess the feasibility of detecting ErrP signals in a single-trial, average ITC of the most prominent peaks of the single-trial failure and success responses of patient 2 were calculated, in the three-time windows W1, W2, and W3. For the failure trials, the average ITC has been observed to be 0.3486, and for the success responses, it has been observed to be 0.4187. To make a comparison, Zhang et al. (2014) reported a maximum ITC 0.36 in their ErrP study involving a monetary gambling task and Yazmir and Reiner (2017) reported a maximum ITC 0.3 in their virtual throwing game ErrP study. It indicates that the novel ErrP signal of this study can be detected in a single-trial with good accuracy using state-of-the-art machine learning methods.

## Discussion

The goal of this study was to check whether an error related potential can be elicited following a failure trial in a post-stroke rehabilitation movement against the success. The obtained results of this study are promising. Overall, it has been found that the ERP, associated with the failure related events and the ERP associated with the success related events, differ from each other significantly and the resulting difference ERP resembles the previously reported ErrP signals and has some unique features which are novel to this new ErrP signal.

In the grand average difference ERP, first positive peak exhibits at 100 ms post the ‘Time’s up!’ screen appeared. After that, a negative peak occurred at 135 ms and finally, a broader negative peak appeared at about 410 ms. These findings are consistent with that of Ferrez and Del R. Millán (2008) who reported a series of positive and negative peaks related to interaction ErrP around 200 to 450 ms post a reference event in a pressing button computer game. A comparison of previous studies has shown that the ErrPs is elicited sooner in the tasks that demand participants to respond quickly and accurately (Vocat et al., 2008; Chavarriaga and Millán, 2010; Ladouceur et al., 2018; Omedes et al., 2018) which is in accordance with the present results. Furthermore, Omedes et al. (2018) also reported a N400 peak in the context of observation of erroneous action in a 3D reaching task, which is consistent with our studies 410 ms negative peak. Prior studies (Olvet and Hajcak, 2008; Weinberg et al., 2015; Rosburg et al., 2018) that have observed the difference ERPs (error/failure minus correct/success trials) noted a negative peak followed by positive peak post-reference event in error trials ERPs. Surprisingly, in this study, reverse polarities have been observed in the failure trial ERP, i.e., a positive peak followed by a negative peak which could be unique to the novel task methodology opted in this experiment. Interestingly, the difference ERP’s final negative peak around 410 ms corroborates the earlier findings (Ferrez and Del R. Millán, 2008; Chavarriaga and Millán, 2010; Omedes et al., 2018). Although the final negative peak around 410 ms popularly, known as N400 is believed to be linked with semantic mismatch (Kutas and Federmeier, 2011), however, N400 existence was also observed during erroneous actions when goal-oriented actions were involved (Balconi and Vitaloni, 2014).

Event-related potentials of both success and failure trials have three prominent peaks. The scalp distribution of the first peak at 100 ms has fronto-central distribution whereas the peaks at 135 and 410 ms have mainly centro-parietal scalp distribution. This finding is consistent with that of Omedes et al. (2018), who also observed fronto-central and centro-parietal scalp distribution of ErrP peaks.

The success events induced power was significant in 3–22, 18–30, 34–38, and 42–50 Hz frequency ranges at various times. On the other hand, the failure events induced power was significant in 11–24, 16–34, and 42–50 Hz at various times. The alpha-band power induced following the success events is in accords with the earlier observations of Carp and Compton (2009), which showed that alpha-band power in the range 8–14 Hz increase and then decrease following a correct response in a six-choice Stroop task; the trend was majorly absent in erroneous response which is consistent with our findings. A possible explanation for this is that in the success response patients completed the exercise before the onset of the ‘Time’s up!’ screen. Therefore, the inter-trial resting interval may have disengaged the patients, and notably, alpha power is linked with the alertness (Carp and Compton, 2009). On the contrary, following the failure response patients’ alertness may have been maintained throughout that caused absence of the quadratic pattern of the induced alpha power. Furthermore, following a failure response, a periodic rebound has been observed in the beta activity that may have been caused due to inhibition of the ongoing motor activity — a trend which has been absent following a success response. A similar activity was observed by Koelewijn et al. (2008) following an erroneous execution of a button-pressing task. It suggests that higher order metal-functions are involved in the evaluation of motor activities even after the execution has stopped.

No significant difference has been observed in the 2- and 4-s failure trials. These findings support our methodology of a direct comparison of 2- and 4-s failure trials taken collectively with the success trials can be made.

### Implications for BCI

In the form of a new task situation that elicits ErrP, the results of this study have demonstrated a clear difference in the failure responses from the success responses. We expect that the results from this study can be transferred to a assist-as-needed (AAN) robot-therapy based rehabilitation program and may be used to improve the current state-of-the-art methods in recognizing the point when the patient has failed in performing the rehabilitation exercise and needed assistance.

The insights gained from this study will also assist developing an adaptive algorithm for AAN methods that can increase/decrease the assistance level on detecting the failure/success related EEG response, respectively. This may make the rehabilitation program continually challenging that may result in an increasingly engaged rehabilitation program that ultimately motivates stroke patients to train or practice longer (Wang et al., 2017).

As the ErrP response associated with the success and the failure events of this study do not demand any mental workload on the participants, therefore, it is expected that the findings of this study can be integrated with existing AAN modalities that may improve the efficacy of the overall rehabilitation program.

### Study Limitations and Future Work

One can argue that the ErrP signals observed in this study are possibly due to the surprise of seeing the finished time-period instead of the failure on executing the rehabilitation exercise. However, it is notable that a surprise forms an integral part of the action-monitoring system of the human brain (Hayden et al., 2011). The experience of error in previous ErrP studies’ tasks such as in gesture controlled robot (Kim et al., 2017), virtual 3D reaching task (Omedes et al., 2018), monitoring cursor on a screen (Chavarriaga and Millán, 2010) had come with a surprise as well. A possible method to reduce this confounding factor is to run the experiment in an asynchronous manner in which the patient will have autonomy of performing rehabilitation exercises, which identifies an area of further research.

Another limitation of the study is the diversity in the participants in terms of days since they suffered a stroke and Brunnstrom stage, which can be possible sources of variability in the EEG signal. The effects of patients’ age, Brunnstrom stage, recovery days, and interest on the amplitude of the ErrP signal elicited in our novel task condition can be studied in the future work.

Another natural progression of this study is to work on detecting the failure and success trials in a single-trial, including against resting periods, so that the ErrP signal can be used in assist-as-needed robot-therapy in real-time. This is an important area for future research.

In EEG studies, reference selection during recording and analysis has always been a source of bias, which can potentially affect the shape of the EEG signals (Murray et al., 2008). Several studies advocate the use of bipolar electrodes to reduce the noise level instead of unipolar references when neural signals only from the cortical sources are of interest (Yao et al., 2019). Several other studies suggest the use of average reference and reference electrode standardization technique (REST) (Yao et al., 2019). Multiple reference techniques should be employed, and their results should be compared to get a reference-bias-free estimate of EEG responses.

## Conclusion

In this paper, we have reported the existence of the error-related potential signal in a new task condition that is when a participant is unable to complete a physical movement in a given time. We conducted experiments on the stroke patients that involved performing a physical rehabilitation exercise. All patients had various levels of limb function impairment. We applied MATLAB and EEGLAB based signal processing techniques to analyze the EEG, EOG, and sEMG data. We used parametric statistical methods to evaluate the statistical significance of the findings. None of the previously reported task conditions that elicit ErrP involve continuous physical exercise movements. To the best of authors’ knowledge, for the first time, an ErrP has been observed while performing rehabilitation exercise. The research has also shown that the reported ErrP has reverse polarity characteristics for the first two peaks of failure trial ERP which is unique to this new task condition ErrP and a final negative peak which is consistent with the results reported in the literature. In summary, the findings reported here shed light on a new type of task that evokes ErrP using which failure events from success events while performing a physical exercise can be distinguished.

## Data Availability Statement

The datasets generated for this study are available on request to the corresponding author.

## Ethics Statement

The experimental protocol was approved by the Ethics Committee of 2nd Hospital of Jiaxing and in accordance with Declaration of Helsinki. All participants were informed about the experiment, and they provided their oral and written consents before the start of the experiment.

## Author Contributions

AK contributed to the experiment design, data collection and analysis, and manuscript preparation. QF contributed to idea formation, experiment design, results discussion, and manuscript preparation. JF and XG contributed to ethics approval, patient recruitment, data collection, and medical guidance. EP contributed to results discussion and manuscript preparation.

## Conflict of Interest

The authors declare that the research was conducted in the absence of any commercial or financial relationships that could be construed as a potential conflict of interest.
